# Impact of rain-shelter cultivation on rhizosphere microecology and kiwifruit quality

**DOI:** 10.3389/fmicb.2025.1600236

**Published:** 2025-06-25

**Authors:** Jianbin Lan, Xixi Dong, Rui He, Qin Huang, Linyu Liu, Junlan Liu, Ailin Tian, Haodan Zhang, Guoqing Sun, Bangzhou Luo, Yinqiu Zeng, Qiang Li

**Affiliations:** ^1^Sichuan Technological Innovation Laboratory for South Subtropical Fruits, College of Agricultural Science, Xichang University, Xichang, China; ^2^Chongqing Key Laboratory for Germplasm Innovation for Special Aromatic Spice Plants, College of Smart Agriculture/Institute of Special Plants, Chongqing University of Arts and Sciences, Chongqing, China

**Keywords:** rain-shelter cultivation, fruit yield, fruit quality, rhizosphere, soil microecology, soil carbon, soil enzyme activity

## Abstract

The effects of kiwifruit rain shelter cultivation on the microecological characteristics of the rhizosphere soil and fruit yield and quality remain uncertain. Therefore, we compared the differences in rhizosphere soil physicochemical properties, microbial populations, enzyme activities, microbial biomass, and fruit yield and quality between kiwifruit rain shelter and open-field cultivation. Additionally, correlations among these parameters were determined. Compared with open-field cultivation, rain-shelter cultivation significantly increased kiwifruit yield (5.17–9.30%), single fruit weight (5.44–6.54%), fruit longitudinal diameter (3.75–4.08%), and transverse diameter (4.58–5.08%), and improved fruit quality, including soluble solids content (9.03–10.05%), soluble sugar content (2.41–4.55%), sugar-to-acid ratio (15.07–20.45%), and vitamin C content (19.03–20.22%). Moreover, rain-shelter kiwifruit cultivation significantly enhanced soil nutrient availability, microbial population, enzyme activities, and electrical conductivity, whereas soil total nutrient and organic matter contents decreased significantly. Further analysis revealed that kiwifruit yield and quality were significantly and positively correlated with available soil nutrients, microbial population, enzyme activities, and microbial biomass carbon and nitrogen but were negatively correlated with the carbon/nitrogen ratio of microbial biomass. These findings indicate that soil microbes and enzymes regulate kiwifruit yield and quality by influencing nutrient availability. Our study provides a firm scientific basis for the efficient soil management and conservation of kiwifruit production, thereby emphasizing the potential of rain shelter cultivation to promote sustainable agriculture.

## Graphical abstract

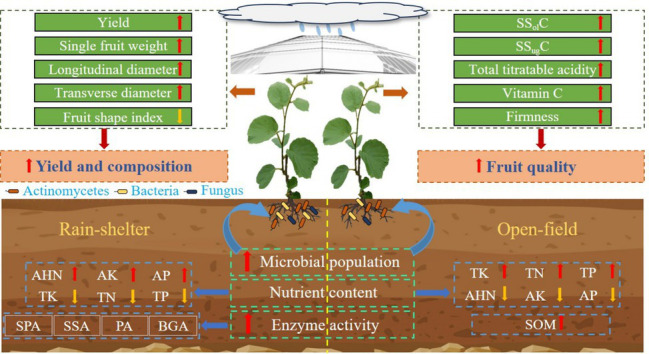

## Highlights

- Rain-shelter cultivation significantly improved kiwifruit fruit yield and quality
- Rain-shelter cultivation enhanced soil nutrient availability
- Rain-shelter cultivation promoted microbial populations and enzyme activities
- Yield and quality correlated positively with soil nutrients and microbial activity
- Microbial biomass C/N ratio negatively correlated with kiwifruit yield and quality

## 1 Introduction

Kiwifruit (*Actinidia* spp.), a perennial woody vine within genus *Actinidia*, is globally renown as the “king of fruits” owing to its rich content of vitamins, minerals, and sugars, whereby it holds an important position in the global fruit market (Wu et al., 2019; Kosta et al., 2023). With its abundant germplasm resources and continuous technological innovations, China has become the largest producer of kiwifruit, with 2.38 million tons produced in 2022, which accounted for 52.4% of total global production (FAO, 2022). However, kiwifruits are highly sensitive to the environment, such that fruit yield and quality are significantly influenced by climate, soil, and crop management (Calderón-Orellana et al., 2021; Gentile et al., 2022; Jiang et al., 2022). Specifically, Chongqing, one of the major kiwifruit-producing regions in China (Wang et al., 2020), frequently faces challenges such as fruit cracking, quality decline, and disease outbreaks due to abundant rainfall after fruit set. These issues have severely hindered the sustainable development of kiwifruit in the region (Miller et al., 1998; Chen et al., 2019; Savian et al., 2020). Therefore, optimizing kiwifruit crop management to enhance adaptability to regional climatic conditions is an urgent necessity and a top research priority.

Rain-shelter (RS) cultivation, which involves constructing rain shelters or covering crops with plastic films, effectively reduces the duration of exposure to rainfall and intense solar radiation. By modifying the field microclimate and improving soil microecological conditions, this practice helps to suppress the spread of plant diseases and ultimately enhances both fruit yield and quality (Huang et al., 2022; Zheng et al., 2023; Shi et al., 2025). For instance, Baiamonte et al. (2016) showed that RS cultivation had little effect on apple maturity, but reduced peel color, sugar content, total phenolic content, volatile compounds, and sensory characteristics. Similarly, Overbeck et al. (2017) reported that RS cultivation not only promoted flowering and accelerated maturity, but enhanced the synthesis of bioactive compounds as well, thereby improving fruit quality. In turn, Chen et al. (2020) reported that RS cultivation increased total sugar, reducing sugar, and sucrose contents in pears, while lowering vitamin C and total acid contents, thus enhancing sweetness and improving fruit quality. Furthermore, Tian et al. (2019) reported that RS cultivation improved single cherry fruit weight and size, reduced the fruit cracking rate, and significantly increased yield per plant. Therefore, RS cultivation not only improves fruit sensory characteristics and nutritional content but, additionally, it reduces losses associated with fruit cracking disease, which makes it an important practice with considerable potential for application in modern agriculture.

Microorganisms are vital components of the soil ecosystem. Their population structure and function directly impact soil health, plant growth, nutrient cycling, and ecosystem responses to climate change (Hartmann and Six, 2023). Certain bacteria and fungi form symbiotic relationships with plant roots. This enhances nutrient and water uptake and produces growth-promoting substances (Das et al., 2022; Khaliq et al., 2022; Adedayo and Babalola, 2023). In addition, microbial numbers are closely linked to soil enzyme activity, which is a key indicator of soil biochemical reactions and fertility (Wang et al., 2023). Understanding and leveraging microorganisms can boost crop productivity and sustainability. This is particularly relevant in RS cultivation, where changes in the microenvironment can significantly affect microbial communities and their functions.

Rhizosphere microbes play crucial roles in plant growth, nutrient absorption, and overall health status (Hardoim et al., 2008). Furthermore, RS cultivation can optimize soil microbial-community structure and significantly increase microbial diversity and abundance, thereby enhancing rhizosphere microenvironment stability (Chen et al., 2020; Zhang et al., 2020). Further, soil enzyme activity and microbial biomass are widely used to assess soil fertility, because they reflect the ecological functions of the soil in response to external stimuli and management practices (Kabiri et al., 2016). A case in point, RS cultivation affects kiwifruit growth, which, in turn, alters the environment of the kiwifruit rhizosphere soil (Lan et al., 2024). Therefore, soil microecological characteristics in the rhizosphere of kiwifruit plants under RS cultivation, especially microbial community structure and soil enzyme activity, may show complex interactions with soil quality and fruit yield.

Previous studies have shown that RS cultivation significantly improves fruit quality, increases yield, and reduces disease-mediated yield losses in crops such as grapes (Yue et al., 2021), strawberries (Claire et al., 2018), peaches (Wu et al., 2015), and pears (Lim et al., 2014). However, little is known about the effects of RS cultivation on soil microecological characteristics, and fruit yield and quality in the case of kiwifruit. Therefore, here, we addressed the following two major questions: (1) How does RS cultivation affect rhizosphere microecology in kiwifruit orchards? (2) What are the relationships between soil microbial biomass, enzyme activity, and fruit yield and quality? Overall, our study aimed to provide sound theoretical and technical support for soil microecology management towards more sustainable development of the kiwifruit industry.

## 2 Materials and methods

### 2.1 Experimental site

Field experiments were conducted in 2022 and 2023 in a kiwifruit orchard located in the Wanzhou District, Chongqing, China (30°24′00″–31°14′58″N, 107°55′22″–108°53′25″E). The study area is part of a subtropical, monsoon humid zone located at the center of the Three Gorges Reservoir, near the central city of the upper Yangtze River. The annual average temperature at the site is 17.7°C; the average annual-sunshine duration is 1484.4 h, and the annual average precipitation is 1,243 mm. The region experiences four distinct seasons with abundant sunlight and rainfall, long frost-free periods, and rare frost or snow events. Kiwifruit is predominantly grown in open field (OF) conditions, where outbreaks of severe bacterial canker diseases are common.

### 2.2 Experimental design

Twelve-year-old ‘Hongyang’ kiwifruit plants cultivated along 40 m long ridges in a north-to-south orientation and with a row spacing of 3 m were used as experimental material. Two treatments were established, RS and OF cultivation. Each treatment included three replicates and 20 robust and uniform plants were tagged in each plot. Fertilization and orchard management followed the standard practices used for kiwifruits of the ‘Hongyang’ cultivar. Soil samples were collected at budding (February 22), leaf expansion (March 17), full-flowering (April 15), maturity (September 5), and leaf fall (October 4) stages using a previously described (Zhu et al., 2021) five-point sampling method. Soil samples were collected within a 20–30 cm radius around the stems of the plants at each sampling site after removing surface debris such as stones and leaves. Rhizosphere soil was collected using the shaking-off method (Riley and Barber, 1969, 1970). Large soil clumps were shaken off and the root-adhering soil was carefully brushed into sterile bags. Samples from the five sampling points were mixed, divided into two portions, and stored in sterile bags. One portion was placed in liquid nitrogen and stored at −80°C for microbial analysis, while the other was air-dried to determine soil physicochemical properties and enzyme activities. Two normal fruits per plant were randomly selected from each plot at maturity (September 5) from five orientations (east, south, west, north, and center) to determine fruit yield and quality parameters.

### 2.3 Determination of fruit quality indexes and yield

#### 2.3.1 Determination of kiwifruit yield quality

Single-fruit weight was measured using an electronic balance (BSA series, Sartorius, Germany), and fruit length and diameter were determined using Vernier calipers (CD-P15S; Mitutoyo Corp., Kanagawa, Japan). The fruit shape index was calculated as described by Dong et al. (2024). In turn, fruit firmness was measured using a GY-1 hardness tester (Sinoyuanda Science and Technology Co., Ltd. Beijing, China), and the soluble solid content (SS_ol_C) was determined using a handheld refractometer (WAY-2S, Dapu Instrument Co., Ltd. Shanghai, China). When fruit firmness reached 1.0–1.2, soluble sugar content (SS_ug_C) was determined using the anthrone colorimetric method. Lastly, titratable acidity (TA) was measured by acid–base titration, and the sugar-to-acid ratio was calculated as in (Duan et al., 2020).

#### 2.3.2 Determination of soil physical and chemical indexes

The soil physicochemical properties were analyzed following the methods described by Bao (2000). Electrical conductivity was measured using the electrode method, soil organic matter (SOM) was determined using the potassium dichromate oxidation-external heating method, total nitrogen (TN) by Kjeldahl digestion, total phosphorus (TP) by NaOH melting and molybdenum-antimony resistance spectrophotometry, and total potassium (TK) by NaOH melting and flame photometry. In turn, alkali-hydrolyzable nitrogen was measured by alkali diffusion, available phosphorus (AP) by ammonium fluoride-hydrochloric acid extraction and molybdenum-antimony colorimetry, and available potassium (AK) by ammonium acetate extraction and flame photometry.

#### 2.3.3 Determination of soil microbial abundance

Soil bacterial, fungal, and actinomycete populations were quantified using the plate-counting method (Zhao and He, 2002).

#### 2.3.4 Determination of soil enzyme activities

Soil protease activity was measured using the colorimetric method (Greenfield et al., 2021); acid phosphatase activity was determined using the p-nitrophenyl disodium phosphate colorimetric method (Tabatabai and Dick, 1979). Meanwhile, *β*-glucosidase activity was measured by the p-nitrophenol colorimetric method (Dick, 2011). The sucrase activity was determined using the 3,5-dinitrosalicylic acid colorimetric method (Duan et al., 2024).

#### 2.3.5 Determination of soil microbial carbon and nitrogen contents

Soil microbial biomass carbon (C) and nitrogen (N) were determined using the chloroform fumigation-extraction method (Brookes et al., 1985; Vance et al., 1987), Organic C in the extracts was measured using a total organic C analyzer (Apollo 9,000 TOC Combustion Analyzer Tekmar-Dohrmann), while total N was analyzed using a continuous flow injection analyzer (Bran-Luebbe AA3).

### 2.4 Data processing and analysis

Statistical analysis for data organization and preliminary calculations was conducted using Microsoft Excel (Office 2021). Differences between treatments were evaluated by *t*-tests using IBM SPSS Statistics version 26 (IBM Corporation, Armonk, NY, United States). Correlation analysis was performed using Pearson’s correlation coefficient values. Graphical visualization of the data was performed using GraphPad Prism version 9 (GraphPad Software, San Diego, CA, USA). All data shown in tables and figures are means ± standard deviation.

## 3 Results

### 3.1 Effects of rain-shelter cultivation on yield and nutritional quality of kiwifruit

Rain-shelter cultivation had highly significant effects on kiwifruit yield and yield components (*p* < 0.01). Indeed, RS cultivation significantly increased kiwifruit yield, and significant differences in single fruit weight and fruit diameter (transverse and longitudinal) were observed relative to OF cultivation (*p* < 0.05); however, no significant effect on fruit shape index was detected (Table 1). Overall, compared to OF, kiwifruit yield under RS increased by 5.17 and 9.30% in 2022 and 2023, respectively. In particular, single-fruit weight increased by 6.54 and 5.44%, fruit transverse diameter increased by 5.08 and 4.58%, and the longitudinal diameter increased by 3.75 and 4.08%, in 2022 and 2023, respectively. Furthermore, compared to OF, the greater increase in transverse than in longitudinal diameter under RS in both years led to a 1.26 and 0.47% decrease in the fruit shape index, respectively.

**Table 1:** Effects of rain-shelter cultivation on yield and yield components of kiwifruit.

Year	Treatment	Yield (t ha^−1^)	Single fruit weight (g)	Transverse diameter (mm)	Longitudinal diameter (mm)	Fruit shape index
2022	RS	18.04 ± 0.40a	75.27 ± 0.33a	47.24 ± 0.35a	58.41 ± 0.28a	1.24 ± 0.01a
	OF	17.15 ± 0.54a	70.65 ± 0.35b	44.95 ± 0.17b	56.30 ± 0.28b	1.25 ± 0.01a
2023	RS	19.99 ± 0.61a	77.70 ± 0.43a	47.98 ± 0.17a	59.04 ± 0.63a	1.23 ± 0.01a
	OF	18.29 ± 0.68b	73.69 ± 0.43b	45.88 ± 0.12b	56.73 ± 0.43b	1.24 ± 0.01a
Year (Y)		**	**	**	ns	**
Treatment (T)		**	**	**	**	**
Y × T		ns	ns	ns	ns	ns

Different lowercase letters in the same column indicate significant differences between treatments (*p* < 0.05). ** denotes highly significant differences at the *p* < 0.01 level, * denotes significant difference between the *p* < 0.05 level, and ns indicates no significant differences. Significant differences were determined using the *t*-test. RS represents rain-shelter cultivation and OF represents open-field cultivation.

Both year and treatment significantly affected kiwifruit quality (*p* < 0.05). Specifically, RS cultivation improved kiwifruit quality, significantly enhancing TSC, SSC, sugar-to-acid ratio, vitamin C content, and fruit firmness (Table 2). Furthermore, compared with OF, under the RS treatment, SS_ol_C increased by 9.03 and 10.05%, SS_ug_C increased by 2.41 and 4.55%, the sugar-acid ratio increased by 15.07 and 20.45%, vitamin C increased by 20.22 and 19.03%, and fruit firmness increased by 6.45 and 5.73%, in 2022 and 2023, respectively. Conversely, relative to OF, the TA content under RS decreased by 11.04 and 13.19% in 2022 and 2023, respectively.

**Table 2:** Effects of rain-shelter cultivation on kiwifruit quality.

Year	Treatment	Soluble solids (%)	Total soluble sugar (%)	Total titratable acidity (%)	Sugar-acid ratio	Vitamin C (mg/100 g)	Fruit firmness
2022	RS	20.28 ± 0.44a	9.92 ± 0.04a	0.89 ± 0.01b	11.19 ± 0.05a	110.47 ± 0.69a	15.19 ± 0.04a
	OF	18.60 ± 0.24b	9.69 ± 0.10b	1.00 ± 0.02a	9.73 ± 0.29b	91.89 ± 0.31b	14.27 ± 0.11b
2023	RS	20.81 ± 0.13a	10.26 ± 0.11a	0.83 ± 0.01b	12.31 ± 0.20a	113.12 ± 0.13a	14.95 ± 0.09a
	OF	18.91 ± 0.13b	9.81 ± 0.04b	0.96 ± 0.01a	10.22 ± 0.09b	95.04 ± 0.34b	14.14 ± 0.06b
Year (Y)		*	**	**	**	**	**
Treatment (T)		**	**	**	**	**	**
Y × T		ns	*	ns	*	ns	ns

Different lowercase letters within the same column indicate significant differences between treatments (*p* < 0.05). ** denotes highly significant differences at *p* < 0.01, * denotes significant differences at *p* < 0.05, and ns indicates no significant differences. Significant differences were determined using a *t*-test.

### 3.2 Effects of rain-shelter cultivation on soil chemical properties of the kiwifruit rhizosphere

The effects of cultivation type on the chemical properties of kiwifruit rhizosphere soil are summarized in Table 3. Compared with OF, RS significantly increased soil electrical conductivity and enhanced alkali-hydrolyzable N, AP, and AK contents. The two-year average electrical conductivity and alkali-hydrolyzable N, AP, and AK increased by 336.32, 25.58, 70.10, and 41.44%, respectively. However, TN, TP, TK, and soil organic matter (SOM) contents significantly decreased by 23.40, 42.11, 20.38, and 14.42%, respectively, under RS over the two experimental years. This finding indicates that, although RS significantly improved soil nutrient availability and electrical conductivity, it reduced soil total nutrient availability and SOM content.

**Table 3:** Effects of rain-shelter cultivation on soil chemical properties.

Year	Treatment	EC (us m^−1^)	TN (g kg^−1^)	TP (g kg^−1^)	TK (g kg^−1^)	AHN (mg kg^−1^)	AP (mg kg^−1^)	AK (mg kg^−1^)	SOM (g kg^−1^)
2022	RS	312.43 ± 4.22a	0.72 ± 0.03b	0.29 ± 0.02b	9.34 ± 0.13b	63.36 ± 1.77a	49.82 ± 1.88a	251.57 ± 3.52a	15.20 ± 0.27b
	OF	72.26 ± 2.92b	0.86 ± 0.04a	0.40 ± 0.03a	10.92 ± 0.10a	54.02 ± 1.38b	30.70 ± 1.32b	189.51 ± 2.71b	17.18 ± 0.32a
2023	RS	334.89 ± 3.45a	0.69 ± 0.02b	0.28 ± 0.02b	9.06 ± 0.09b	66.82 ± 3.14a	52.68 ± 1.93a	265.51 ± 2.54a	14.75 ± 0.10b
	OF	76.10 ± 2.73b	0.88 ± 0.03a	0.41 ± 0.02a	11.23 ± 0.13a	49.64 ± 1.52b	29.56 ± 1.67b	176.06 ± 1.67b	17.09 ± 0.15a
Year (Y)		**	ns	ns	ns	ns	ns	ns	ns
Treatment (T)		**	**	**	**	**	**	**	**
Y × T		**	ns	ns	**	*	ns	**	ns

Different lowercase letters within the same column indicate significant differences between treatments (*p* < 0.05). ** denotes highly significant differences at *p* < 0.01, * denotes significant differences at *p* < 0.05, and ns indicates no significant differences. Significant differences were determined using a *t*-test.

### 3.3 Effects of rain-shelter cultivation on soil microbe abundance in kiwifruit rhizosphere

**Figure 1:**
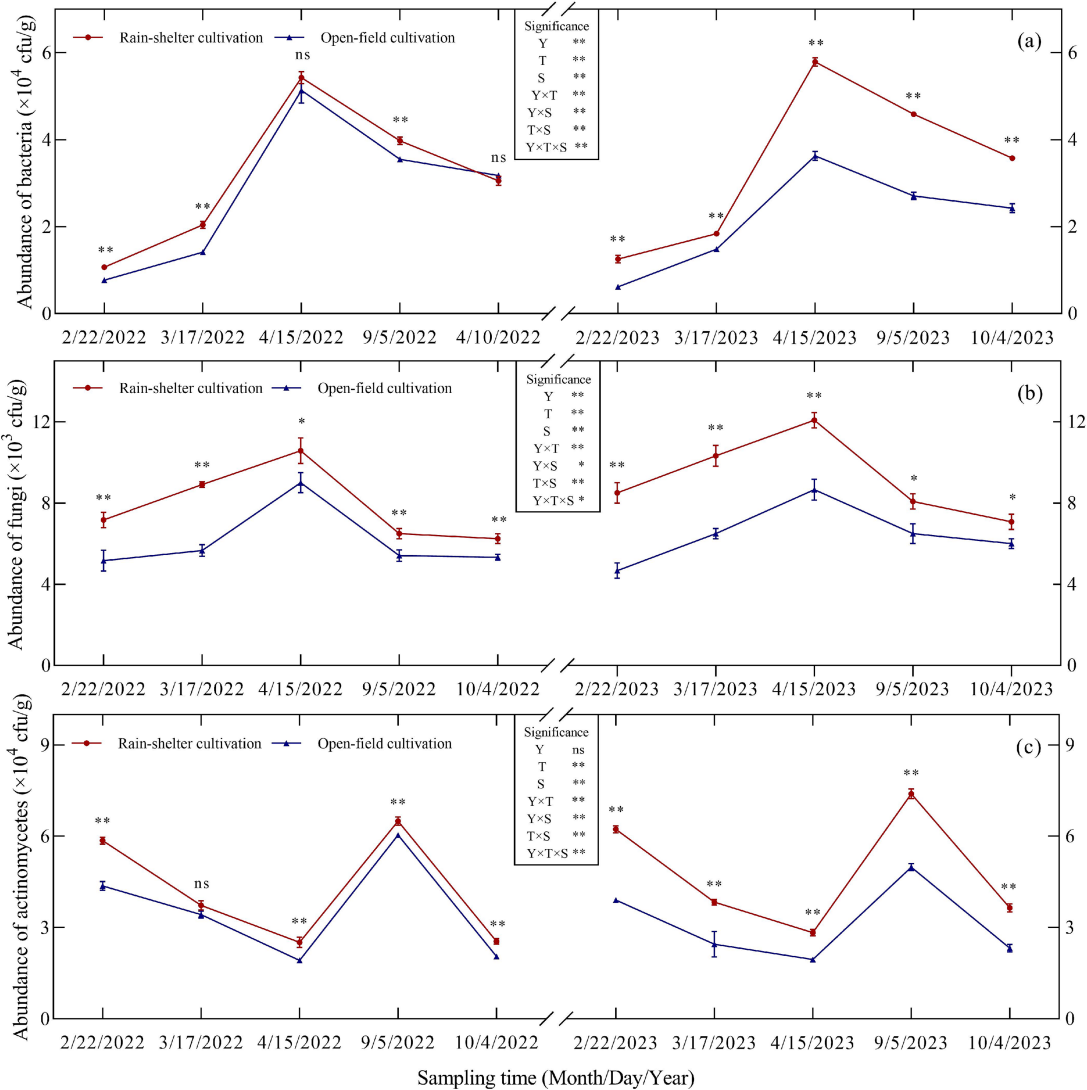
Abundance of microorganisms in the rhizosphere soil of kiwifruit trees grown under rain-shelter and open field cultivation conditions. **(a)** Abundance of bacteria. **(b)** abundance of fungi. **(c)** abundance of actinomycetes. Y represents Year, T represents Treatment, and S represents the Sampling Time. Significant differences were determined using a t-test; ns represents no significance, * *p* < 0.05, ** *p* < 0.01.

Year, treatment, and sampling time, all had highly significant effects on the active microbial populations in the kiwifruit rhizosphere soil (*p* < 0.01); additionally, our analysis revealed significant interaction effects (Figure 1). Actinomycetes dominated the active microbial population in the rhizosphere, followed by bacteria and fungi. Rain-sheltered cultivation increased active microbial populations in the rhizosphere soil at different stages (except for the bacterial count in the leaf-fall stage in 2022). In particular, bacterial populations under RS and those observed under OF cultivation differed significantly (*p* < 0.01; Figure 1a) during budding, leaf expansion, and maturity but not during the flowering or leaf-fall stages. Similarly, RS cultivation differed significantly from OF (*p* < 0.05; Figure 1b) with respect to fungal populations. In particular, a significant difference was observed between the two treatments with respect to actinomycetes at all stages except for the leaf expansion stage in 2022 (*p* < 0.05; Figure 1c). In general, as the growing season progressed, bacterial and fungal abundances initially increased and then decreased, peaking at full flowering. Thus, compared with OF, under RS cultivation, bacterial abundance increased by 5.71 and 59.56%, whereas fungal abundance increased by 17.59 and 39.42%, respectively. In turn, actinomycete abundance initially decreased and then increased, peaking during maturity, with increases of 7.48 and 48.35%, respectively, compared to OF, and then decreased again. These results suggest that RS cultivation increased the overall abundance of active microorganisms in the kiwifruit rhizosphere and consequently improved the microecosystem.

### 3.4 Effects of rain-shelter cultivation on soil enzyme activity in the rhizosphere soil of kiwifruit trees

**Figure 2:**
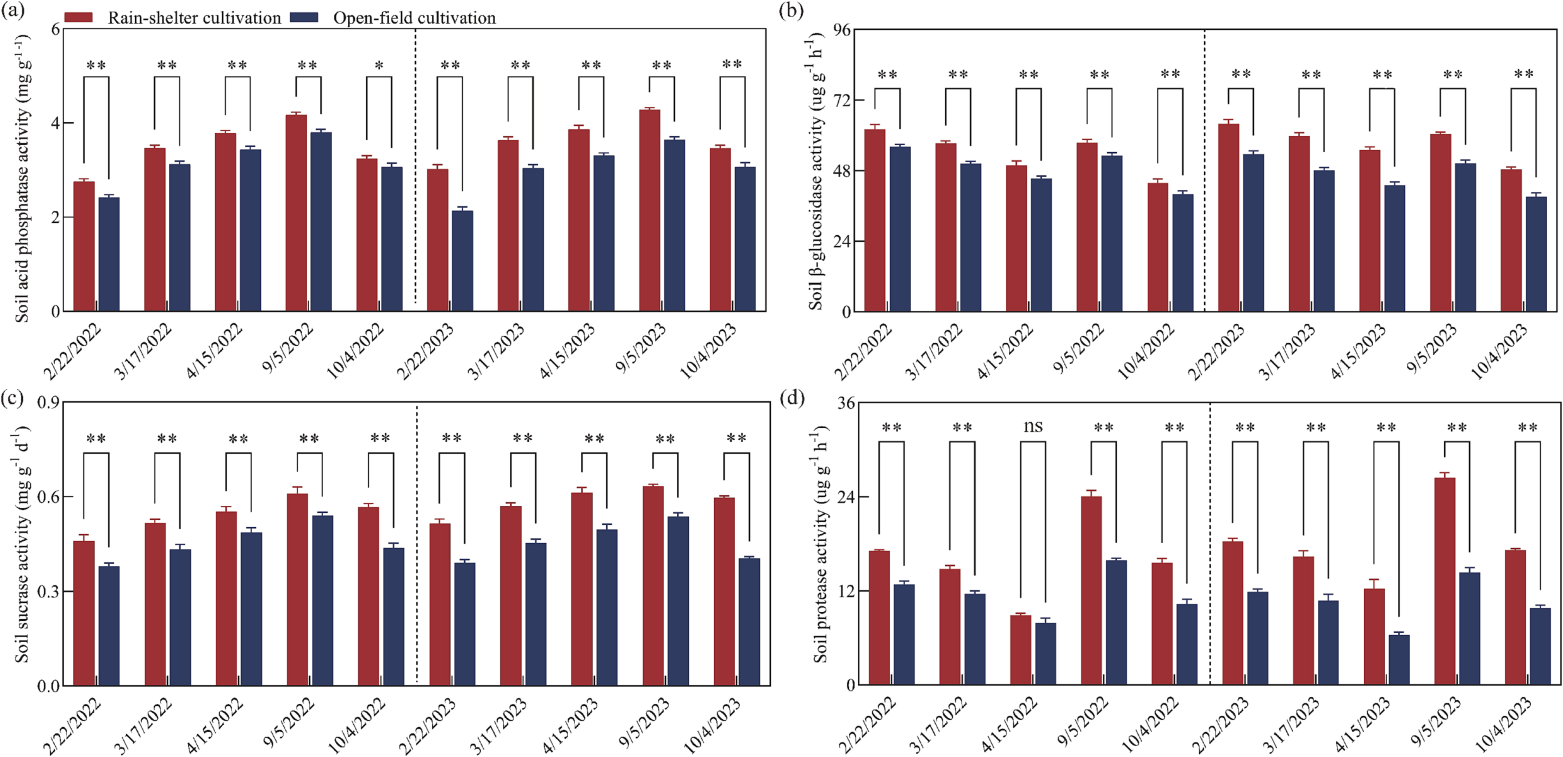
Enzyme activities in the rhizosphere soil of kiwifruit trees grown under rain-shelter and open field conditions. **(a)** Acid phosphatase, **(b)** *β*-glucosidase, **(c)** sucrase, and **(d)** protease. Significant differences were determined using the *t*-test; ns represents no significance, * *p* < 0.05, ** *p* < 0.01.

Acid phosphatase and sucrose metabolism-related enzyme activities in the rhizosphere soil under the RS and OF treatments initially increased and then continuously decreased over the two experimental years. In general, RS cultivation significantly enhanced the activities of acid phosphatase and sucrose enzymes at different stages (*p* < 0.05), with the highest activity recorded at maturity (Figures 2a,c). Thus, compared to OF, RS cultivation increased the activity of acid phosphatase by 5.66 and 13.93%, and by 12.84 and 41.19%, and those of sucrose metabolism-related enzyme activities by 12.96 and 29.77%, and by 18.01 and 47.93%, in 2022 and 2023, respectively.

In contrast to the enzyme activities mentioned above, the activities of *β*-glucosidase and protease in the rhizosphere soil under the two treatments first decreased, then increased, and finally decreased again over the two experimental years. In particular, RS cultivation significantly enhanced *β*-glucosidase and protease activities at the different developmental stages (*p* < 0.05), except at the full-flowering stage in 2022. Further, the highest β-glucosidase activity was recorded during the budding stage (Figure 2b), while that of the protease activity was recorded at maturity (Figure 2d). Overall, compared to OF, RS cultivation increased the activity of β-glucosidase by 8.29 and 13.72%, by 19.24 and 24.19%, and protease activity by 12.93 and 51.12%, and 52.34 and 92.68%, in 2022 and 2023, respectively.

### 3.5 Effects of rain-shelter cultivation on soil microbial-biomass C and N in the rhizosphere soil of a kiwifruit tree orchard

**Figure 3:**
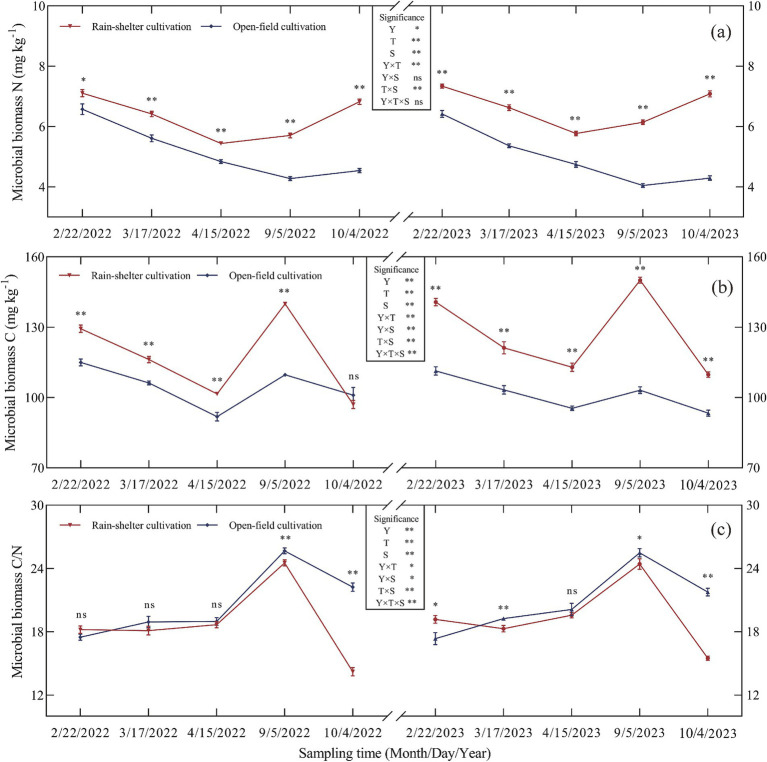
Microbial biomass of kiwifruit rhizosphere soil under rain-shelter and open field cultivation. **(a)** Microbial biomass N, **(b)** Microbial biomass C, and **(c)** Microbial biomass C/N. Y represents Year, T represents Treatment, and S represents Sampling Time. Significant differences were determined using the *t*-test, ns represents no significance, * *p* < 0.05, ** *p* < 0.01.

Year, treatment, and sampling time, all had significant effects on microbial biomass carbon (MBC), microbial biomass nitrogen (MBN), and the microbial biomass C/N ratio in the kiwifruit rhizosphere (*p* < 0.05, Figure 3). Particularly, RS cultivation increased MBN (Figure 3a). During the growing season, MBN in the rhizosphere soil first decreased and then increased, with peak values observed during budding. Compared with OF, MBN was 8.11 and 14.33% higher at the same stage in both experimental years, with the differences being significant (*p* < 0.05).

In addition to the leaf abscission stage in 2022, RS cultivation also increased MBC in the rhizosphere soil. Over the 2 years, MBC first decreased, then increased, and finally decreased again, with the highest values observed at maturity. Compared with OF, MBC increased by 37.60 and 45.43% at the same stage in 2022 and 2023, respectively; furthermore, the differences were highly significant (*p* < 0.01; Figure 3b).

The microbial C/N ratio initially increased and then decreased over the 2 years, with the highest values observed at maturity. Compared to OF, the microbial C/N ratio decreased by 4.45 and 4.18% at the same stages in 2022 and 2023, respectively, with significant differences (*p* < 0.05) recorded at maturity and leaf abscission (Figure 3c).

**Figure 4:**
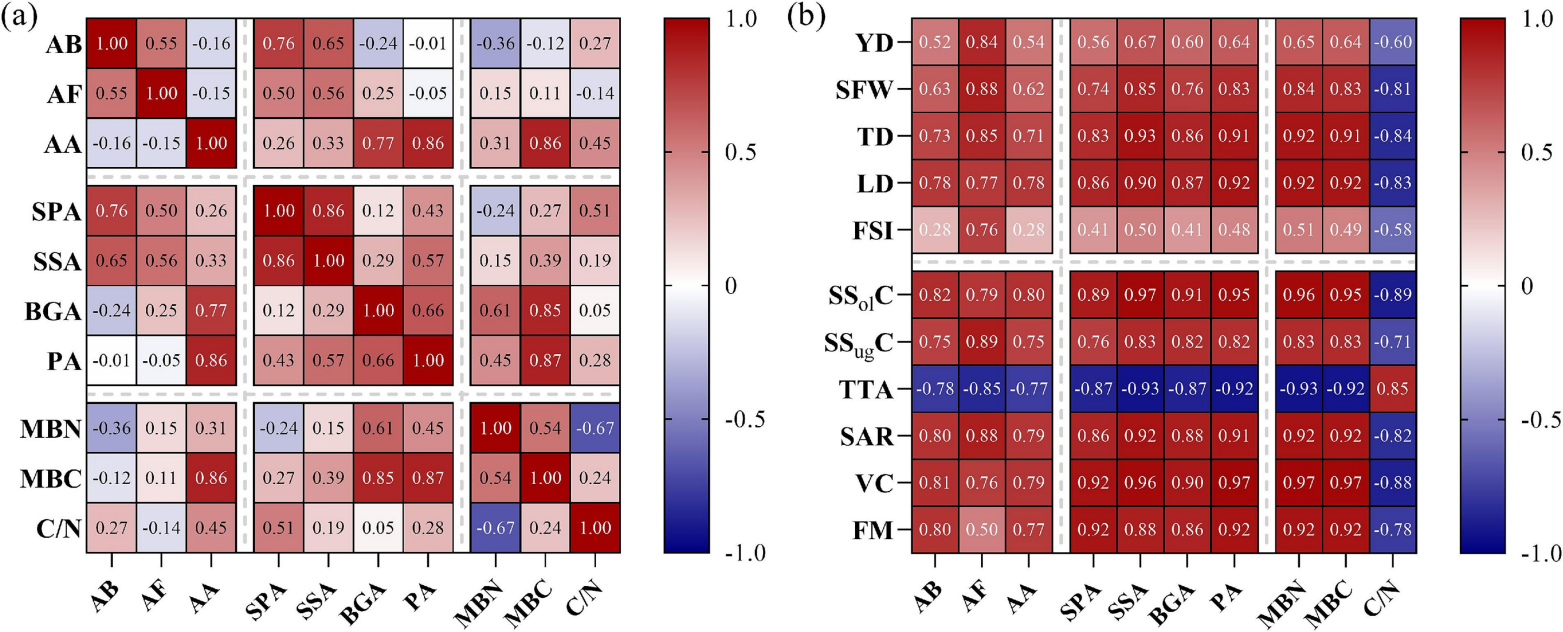
Correlation analysis between kiwifruit rhizosphere-soil microbial indicators, enzyme activities, and yield and quality traits. **(a)** Microbial indicators and enzyme activities. **(b)** yield and quality traits of kiwifruit. AB (abundance of bacteria), AF (abundance of fungi), AA (abundance of actinomycetes), SPA (soil acid phosphatase activity), SSA (soil sucrase activity), BGA (soil β-glucosidase activity), PA (soil protease activity), MBN (microbial biomass nitrogen), MBC (microbial biomass carbon), C/N (microbial biomass carbon-to-nitrogen ratio), YD (yield), SFW (single fruit weight), TD (transverse diameter), LD (longitudinal diameter), FSI (fruit shape index), SS_ol_C (soluble solids), SS_ug_C (total soluble sugars), TTA (titratable total acids), SAR (sugar-acid ratio), VC (vitamin C), and FM (fruit firmness).

Rhizosphere soil-microbial indicators, enzyme activities, and kiwifruit yield and quality (Figure 4) were correlated to varying degrees. Thus, bacterial and fungal abundances were significantly and positively correlated with acid phosphatase and sucrase activities and with the soil microbial biomass C/N ratio. Conversely, they were significantly and negatively correlated with MBN. In turn, actinomycete abundance was significantly and positively correlated with sucrase, *β*-glucosidase, protease, MBC, microbial biomass C/N ratio, acid phosphatase, and MBN. Furthermore, acid phosphatase activity was significantly and positively correlated with the microbial biomass C:N ratio and MBC. Similarly, sucrase activity significantly and positively correlated with MBC. Meanwhile, β-glucosidase and protease activities highly significantly and positively correlated with MBC and MBN. Moreover, protease activity was significantly and positively correlated with the microbial biomass C/N ratio. In turn, MBN was significantly and positively correlated with MBC, but significantly and negatively correlated with the microbial biomass C/N ratio (Figure 4a).

Kiwifruit yield correlated highly significantly and positively with fungi abundance, sucrase, β-glucosidase and protease activities, and with MBC and MBN, but significantly and negatively with the microbial biomass C/N ratio. Similarly, single-fruit weight was significantly and positively correlated with fungal abundance, soil enzyme activities, MBC and MBN, and with bacterial and actinomycete abundances, which were significantly and negatively correlated with the microbial biomass C/N ratio. Additionally, fruit transverse diameter, longitudinal diameter, SS_ol_C, SS_ug_C, sugar-to-acid ratio, and vitamin C content were significantly and positively correlated with microbial abundance, enzyme activities, MBC, and MBN but significantly and negatively correlated with the microbial biomass C/N ratio. Lastly, TA content was significantly and negatively correlated with microbial abundance, enzyme activities, MBC, and MBN, but significantly and positively correlated with the microbial biomass C/N ratio (Figure 4b).

## 4 Discussion

Fruit yield and quality are important indicators of the effectiveness of plant cultivation and are closely related to ecological conditions and crop management (Atmaca et al., 2024). Lan et al. (2024) reported that RS cultivation significantly increased soil nutrient availability for kiwifruit, thereby enhancing crop yield. Consistently, Liu et al. (2022) found that single-fruit weight and fruit diameter (both transverse and longitudinal) increased significantly during maturity, although the fruit shape index did not differ significantly. Similarly, the results of this study showed that, compared with OF, RS cultivation significantly increased yield, single fruit weight, and both longitudinal and transverse diameters (Table 1). Furthermore, although the fruit shape index decreased slightly over the 2 years, the difference was not significant. These findings are also consistent with those of Zeng et al. (2020) and Lin et al. (2016) for pears, indicating that RS cultivation effectively increased fruit transverse and longitudinal diameters and, consequently, fruit yield. Further, fruit quality is one of the most important criteria for assessing marketability. With respect to this issue, our results indicate that, compared with OF, RS cultivation significantly improved SS_ol_C, SS_ug_C, sugar-to-acid ratio, vitamin C content, and fruit firmness, while significantly reducing titratable acidity (Table 2). These findings suggest that RS cultivation not only improved kiwifruit sweetness, but reduced acidity as well, further confirming previous studies that RS cultivation improves fruit quality (Tian et al., 2019; Chen et al., 2020).

Soil physicochemical characteristics provide key parameters for assessing soil fertility and health. Furthermore, changes in cultivation practices often lead to significant variations in these properties. Our results showed that, compared with OF, RS cultivation significantly increased soil nutrient availability (alkali-hydrolyzable N, AP, and AK) and electrical conductivity, while total nutrients (TN, TP, and TP) and SOM content significantly decreased (Table 3). These results are consistent with findings reported by Chen et al. (2020), and are primarily explained by the accelerated decomposition of SOM and reduced nutrient loss caused by the larger microbial populations promoted under RS cultivation, which in turn improve nutrient availability. Additionally, soil electrical conductivity significantly increased under RS cultivation, reflecting an increase in soil-soluble ion concentrations and salinization, which is consistent with the results of Li et al. (2015). Therefore, although RS cultivation can improve soil nutrient supply to some extent, there is a potential risk of soil nutrient imbalance and salinization in the long term. Integrated soil-management strategies are essential to mitigate these adverse effects and ensure sustainable soil fertility. For instance, incorporating compost or organic amendments can help replenish SOM and buffer nutrient loss (Singh et al., 2024). In turn, the use of cover crops can reduce nutrient leaching and improve soil structure, while crop rotation can enhance microbial diversity and nutrient cycling (Ding et al., 2024). Altogether, these strategies contribute to the maintenance of a balanced soil ecosystem under RS cultivation schemes, thereby ensuring long-term productivity and ecological sustainability.

Soil microorganisms and enzyme activities are important indicators of soil fertility because they play a crucial role in the flow of energy and material cycling within the soil (Sun et al., 2009; Hu et al., 2023). The results of this study showed that, compared with OF cultivation, bacterial, fungal, and actinomycete abundances in the kiwifruit rhizosphere soil were significantly higher under RS cultivation, with actinomycetes being the most abundant (Figure 1), mainly due to the light and dry soil environment under such conditions, which favored the growth of actinomycetes, a finding that is consistent with those of Lin et al. (2018) indicating that RS cultivation improved the rhizosphere environment and increased microbial populations. Moreover, compared with OF cultivation, soil enzyme activities, including acid phosphatase, sucrase, *β*-glucosidase, and protease, were significantly higher under RS cultivation at different developmental stages (Figure 2), with acid phosphatase and protease showing larger increases than the other two enzymes. These results are consistent with those of Chen et al. (2020) suggesting that RS cultivation effectively enhanced soil enzyme activity, although there were significant differences in enzyme types, likely related to nutrient utilization in the soil. Further analysis revealed that MBC and MBN in the rhizosphere soil of kiwifruit trees were significantly higher under RS cultivation, whereas the microbial biomass C/N ratios were significantly reduced (Figures 3a,b). These findings are consistent with those of Li et al. (2022) and Luo et al. (2021), and indicate that RS cultivation improved the soil microenvironment, enhanced microbial metabolic activity, and contributed significantly to an increase in soil nutrient availability (alkali-hydrolyzable N, AP, and AK). Additionally, during leaf abscission, MBC decreased significantly, whereas MBN increased, suggesting that at this stage, microbial metabolism prioritized the utilization of C sources. Further, correlation analysis showed a significant positive correlation between soil microbial populations and acid phosphatase and sucrase activities, and between soil enzyme activities (acid phosphatase, sucrase, β-glucosidase, and protease) and MBC, which is consistent with results reported by Zhang et al. (2020). The abundance of actinomycetes was significantly and positively correlated with soil MBC, MBN, and the MB C/N ratio (Figure 4a), indicating that the increase in microbial populations enhanced the soil storage capacity for organic C and N. Furthermore, fruit yield and quality (sugar-to-acid ratio, soluble solids, and vitamin C content) significantly and positively correlated with soil nutrient availability, microbial populations, enzyme activities, and MBC and MBN, while showing a significant negative correlation with microbial biomass C/N ratio (Figure 4b). Consistent with previous reports (Chen et al., 2020; Zhang et al., 2020), the results summarized herein strongly indicate that soil microbial populations and enzyme activities regulate kiwifruit yield and quality by influencing soil nutrient supply.

## 5 Conclusion

Soil nutrient availability, electrical conductivity, microbial populations, enzyme activities, MBC, and MBN significantly increased under RS cultivation, whereas total soil nutrients, SOM, and microbial biomass C/N ratio significantly decreased. Concomitantly, kiwifruit yield, single-fruit weight, and transverse and longitudinal fruit diameters significantly increased, while fruit quality (SS_ol_C, SS_ug_C, sugar-acid ratio, and vitamin C) was significantly enhanced. Further analysis revealed that fruit yield and quality were significantly and positively correlated with soil nutrient availability, microbial populations, enzyme activities, MBC, and MBN, but significantly and negatively correlated with the microbial biomass C/N ratio. These results suggest that soil microorganisms and enzymes play crucial roles in kiwifruit growth, development, and quality by regulating soil nutrient supply and metabolic processes. However, these conclusions are based on correlation analysis rather than direct evidence of causality. The underlying microbial functional mechanisms, such as shifts in the microbial community structure or functional gene expression, require validation in future studies that use approaches such as microbial sequencing or metagenomics. Additionally, although we observed improvement in basic fruit-quality indicators, sensory traits such as flavor, aroma, and texture, which directly influence marketability and consumer acceptance, were not assessed in this study. Thus, future research should incorporate sensory evaluations to provide a more comprehensive assessment of fruit quality. Finally, although RS cultivation improved short-term nutrient availability, the observed decrease in total nutrients and SOM, concomitant with the increase in electrical conductivity, indicate a potential risk of long-term soil degradation or salinization. To minimize such risk, future cultivation strategies should incorporate organic amendments, cover crops, and crop rotation schemes to maintain soil health and sustainability. Overall, RS cultivation remains a promising approach for enhancing kiwifruit production; however, its broader agroecological implications must be thoroughly considered to provide a solid theoretical foundation for the design of the best management strategies.

## Data Availability

The raw data supporting the conclusions of this article will be made available by the authors upon reasonable request.
